# From psoriasis to psoriatic arthritis: epidemiological insights from a retrospective cohort study of 74,046 patients

**DOI:** 10.3389/fmed.2024.1419722

**Published:** 2024-06-27

**Authors:** An-Ping Huo, Pei-Lun Liao, Pui-Ying Leong, James Cheng-Chung Wei

**Affiliations:** ^1^Institute of Medicine, Chung Shan Medical University, Taichung, Taiwan; ^2^School of Medicine, Chung Shan Medical University, Taichung, Taiwan; ^3^Division of Allergy, Immunology and Rheumatology, Department of Internal Medicine, Chung Shan Medical University Hospital, Taichung, Taiwan; ^4^Department of Medical Research, Chung Shan Medical University Hospital, Taichung, Taiwan; ^5^Department of Nursing, Chung Shan Medical University, Taichung, Taiwan; ^6^Graduate Institute of Integrated Medicine, China Medical University, Taichung, Taiwan; ^7^Office of Research and Development, Asia University, Taichung, Taiwan

**Keywords:** psoriasis, psoriatic arthritis, TriNetX, smoking, diabetes mellitus

## Abstract

**Introduction:**

To verify our hypothesis that psoriatic arthritis (PsA) is mainly genetically predetermined and distinct from psoriasis (PsO), we use the TriNetX database to investigate whether intrinsic factors outweigh externals in PsA emergence in PsO patients.

**Methods:**

We conducted three retrospective cohort studies utilizing information from the TriNetX network, whether (a) PsO patients with type 2 diabetes mellitus (DM) face an elevated risk of developing PsA compared to those without type 2 DM; (b) PsO patients who smoke face a higher risk of PsA; and (c) PsO patients with type 2 DM who smoke are more likely to develop PsA than those who do not smoke.

**Results:**

PsO patients with type 2 DM exhibited an elevated risk of developing PsA [hazard ratio (HR), 1.11; 95% CI 1.03–1.20], with the combined outcome demonstrating a heightened HR of 1.31 (95% CI 1.25–1.37). PsO patients with a smoking history exhibited an elevated risk of developing PsA (HR, 1.11; 95% CI 1.06–1.17), with the combined outcome demonstrating a heightened HR of 1.28 (95% CI 1.24–1.33). PsO patients with type 2 DM and a history of smoking were not found to be associated with an increased risk of developing PsA (HR, 1.05; 95% CI 0.92–1.20). However, the combined result revealed a higher risk of 1.15 (95% CI 1.06).

**Discussion:**

These findings suggested that intrinsic factors outweigh external factors in PsA emergence in PsO patients. Further studies may focus on genetic disparities between PsO and PsA as potential risk indicators rather than solely on phenotypic distinctions.

## Introduction

Psoriatic arthritis (PsA) is a persistent inflammatory condition characterized by various musculoskeletal symptoms, such as peripheral arthritis, dactylitis, enthesitis, and spondylitis, linked to a history of psoriasis (PsO) or ongoing PsO disease. The reported incidence rate of PsA in individuals with PsO ranges from 6 to 41% (1). Approximately 70% of PsA cases are diagnosed around 9–10 years after the initial diagnosis of PsO, while 20% are diagnosed simultaneously with PsO. Less than 10% of PsA cases are identified without PsO lesion (2). With advancements in PsO treatment, including new biologic disease-modified anti-rheumatic drugs (bDMARDs), recent evidence suggests that using either biologics or conventional synthetic disease-modified anti-rheumatic drugs (csDMARDs) for PsO can reduce the likelihood of developing PsA (3–5). Animal models indicate that persistent interleukin (IL)-23 stimulation may trigger PsO, and its continuation may lead to the development of PsA, suggesting IL-23 as a potential pathogenic initiator for both conditions (6). Some studies propose that the Psoriasis Area and Severity Index (PASI) and body mass index (BMI) as external factors may influence the transition from PsO to PsA (7). Consequently, early intervention in PsO is recommended to prevent the onset of PsA, particularly with the use of enhanced bDMARDs treatment for PsO (8).

Nevertheless, approximately 5–10% of PsA patients experience musculoskeletal manifestations before the appearance of psoriasis skin lesions (9). Despite using advanced biologics in PsO treatment, PsA can still manifest, even with aggressive PsO treatments (10–14). Considering the transition from PsO to PsA, various endeavors have been undertaken in clinical, genetic, epigenetic, imaging abnormality, cellular, tissue, and molecular markers to identify potential risk factors for PsO patients progressing to PsA. However, the results of these efforts remain somewhat disappointing (15).

The prevailing viewpoint considers PsO and PsA manifestations of the same disease with distinct phenotypes, and it is commonly believed that PsA represents a progression from PsO. However, from our clinical observations and the inconsistent evidence presented above, we considered that PsA and PsO share similar but not identical genetic backgrounds, leading to different signaling pathways and resulting in distinct phenotypes. We liken this relationship to that of brothers, not twins. Contrary to the notion that PsA develops as a transition from PsO, we proposed that the intrinsic factors outweigh externals in PsA emergence in PsO patients, not a sequential progression from PsO. Given the known genetic connection between PsO and metabolic conditions such as type 2 diabetes mellitus (DM) (16–18), we chose type 2 DM as an intrinsic factor to see its impact on PsA emergence in PsO patients.

On the other hand, considering smoking as an external trigger documented to worsen Psoriasis Area Severity Index (PASI) scores (19, 20). And is thought to increase the risk of PsA onset in PsO patients (21), we chose smoking as an external factor to see its influence on PsA development in PsO patients. To access our hypotheses, we utilized the TriNetX database to investigate whether (a) PsO patients with type 2 DM face an elevated risk of developing PsA compared to those without type 2 DM; (b) PsO patients who smoke face a higher risk of PsA; and (c) PsO patients with type 2 DM who smoke are more likely to develop PsA than those who do not smoke.

## Methods

### Data source

Two retrospective cohort studies utilized information from the TriNetX network (https://trinetx.com/). TriNetX is a global health research network that provides access to de-identified electronic medical records, covering diagnoses, procedures, medications, laboratory test results, and genomic information. We obtained data from a subset of the database involving 56 healthcare organizations in the United States. The data analysis was executed in September 2023. This dataset has been validated in many previous studies (3, 22). The TriNetX platform uses aggregated counts and statistic summaries of de-identified information, and no protected health information or personal data is available to the platform users; therefore, after consulting the local institutional review board, there is no need for ethical approval on studies using the TriNetX health research network.

### Study population

This retrospective cohort study spanned from January 1, 2012, to December 30, 2022. The first cohort included individuals diagnosed with PsO using the ICD-10-CM code L40 within this timeframe. The variable of interest was type 2 diabetes, identified through ICD-10-CM codes E08 and E11. The study cohort comprised individuals with PsO with type 2 DM, while the control group consisted of PsO patients without type 2 DM.

Subsequently, the second cohort included individuals diagnosed with PsO based on ICD-10-CM code L40 during the same period. The variable of interest was smoking, which was identified by the ICD-10-CM codes Z72.0 and F17. The study cohort included individuals with both PsO and smoking history, while the control group encompassed PsO patients who were non-smokers.

We additionally grouped individuals with PsO and type 2 DM to compare the risk of PsA with and without smoking.

### Outcome and covariates

The primary outcome involved the identification of PsA (ICD-10-CM codes L40.5). Second, we combined each outcome with death in a composite measure to address competing risks and survivorship bias. The study encompassed a range of covariates considered within 1 year preceding the index date. Demographic variables included age at the index date, sex, ethnicity, and race. Medical utilization was evaluated by assessing Office or Other Outpatient Services (CPT: 1013626) and Hospital Inpatient Services (CPT: 1013659). Socioeconomic and psychosocial aspects were explored using codes associated with issues in education and literacy (Z55), employment and unemployment (Z56), occupational exposure to risk factors (Z57), and housing and economic circumstances (Z59). Lifestyle factors, such as tobacco use (ICD-10-CM codes Z72.0), nicotine dependence (ICD-10-CM codes F17), and alcohol-related disorders (ICD-10-CM codes F10), were considered. Additionally, various comorbidities were included in the analysis, such as hypertensive diseases (ICD-10-CM codes I10-I16), hyperlipidemia (ICD-10-CM codes E78), asthma (ICD-10-CM codes J45), COPD (ICD-10-CM codes J44), atrial fibrillation and flutter (ICD-10-CM codes I48), heart failure (ICD-10-CM codes I50), mental/dementia (ICD-10-CM codes F01-F09), chronic kidney disease (ICD-10-CM codes N18), anxiety disorders (ICD-10-CM codes F41), and diseases of the liver (ICD-10-CM codes K70-K77). These comprehensive variables were analyzed to uncover potential associations and impacts within the study population.

### Statistical analysis

To mitigate the impact of confounding variables, we employed TriNetX's built-in function to create groups with matched baseline characteristics through 1:1 propensity score matching. The standardized mean difference (SMD) was utilized to assess the balance of baseline characteristics in the propensity score-matched groups. SMD values below 0.10 serve as an indicator of balance within the studied population.

Cox proportional hazards regression analysis was conducted to compare paired cohorts, presenting hazard ratios and 95% confidence intervals (95% CI). The incidence of psoriatic arthritis was determined using the Kaplan-Meier method, and the log-rank test was applied. Statistical significance was defined as a *p*-value < 0.05.

## Results

### Baseline characteristics of the study population

We have presented baseline demographic data for various groups in two retrospective cohorts, as outlined in Supplementary Tables 1, 2. After applying Propensity Score Matching (PSM) to the first cohort, we identified 42,315 patients in the PsO with type 2 DM groups and the PsO without type 2 DM groups. Notably, the PsO group included no patients with type 2 DM. In the second cohort post-PSM, we identified 74,046 patients with a history of smoking in the PsO group and an equal number of 74,046 patients without a history of smoking in the PsO group. The demographic distributions and comorbidities exhibited similarities between the study and control groups, with standardized differences consistently below 0.1.

### Psoriasis and type 2 diabetes patients face a higher risk of psoriatic arthritis

**Figure 1:**
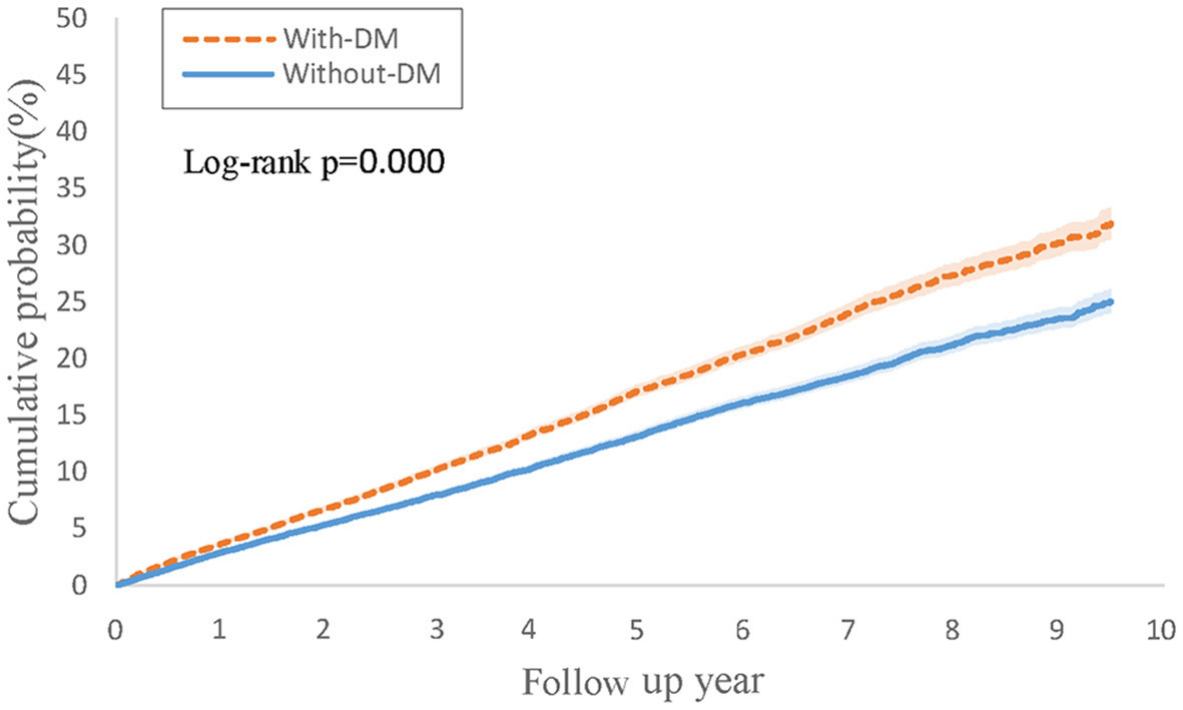
Kaplan-Meier curves of cumulative probability (%) of psoriatic arthritis comparing psoriasis with type 2 diabetes and without type 2 diabetes groups.

Table 1 illustrates the findings from Cox regression analysis for the initial group. In comparison to patients with PsO but without type 2 DM, those with both PsO and type 2 DM exhibited an elevated risk of developing PsA (HR, 1.11; 95% CI 1.03–1.20), with the combined outcome demonstrating a heightened hazard ratio of 1.31 (95% CI 1.25–1.37). Figure 1 shows the Kaplan-Meier curves revealed a higher incidence of PsA in the group with both PsO with type 2 DM compared to the group without type 2 DM (log-rank *P* < 0.001).

**Table 1:** Hazard ratio and 95% CIs for the risk of psoriatic arthritis.

	**Patients in cohort**	**Patients with outcome**	Hazard ratio^*^(95% CI)
**Composite End Points**			
**Risk of psoriatic arthritis**			
Psoriasis with type 2 diabetes	42,315	1,281	1.11 (1.03, 1.20)
Psoriasis without type 2 diabetes	42,315	1,296	Reference
**Risk of psoriatic arthritis or mortality**			
Psoriasis with type 2 diabetes	42,315	4,245	1.31 (1.25, 1.37)
Psoriasis without type 2 diabetes	42,315	3,708	Reference

^*^Hazard ratio for outcomes among Psoriasis with type 2 diabetes group compared to Psoriasis without type 2 diabetes group subjects (after prosperity score matching).

95% CI, 95% confidence interval.

Upon conducting a stratified analysis, it was observed that females with PsO and type 2 DM faced an increased risk of developing PsA compared to PsO patients without type 2 DM (HR, 1.27; 95% CI 1.14–1.41), as detailed in Table 2. We additionally conducted a sensitivity analysis utilizing the Global Collaborative Network (Supplementary Table 3), and the findings remained consistent. In comparison to patients with PsO but without type 2 DM, those with both PsO and type 2 DM exhibited an elevated risk of developing PsA (HR, 1.09; 95% CI 1.01–1.18), with the combined outcome demonstrating a heightened hazard ratio of 1.32 (95% CI 1.26–1.38).

**Table 2:** Hazard ratio and 95% CIs for the risk of psoriatic arthritis by stratified analysis.

	**Patients with outcome**		Hazard ratio^*^(95% CI)
	**Psoriasis with type 2 diabetes**	**Psoriasis without type 2 diabetes**	
**Composite End Points**			
**Risk of psoriatic arthritis**			
**Sex**			
Female	732	633	1.27 (1.14–1.41)
Male	538	624	0.98 (0.87, 1.10)
**Age**			
≤ 50	460	502	1.03 (0.91, 1.17)
>51	887	965	1.01 (0.92, 1.10)
**Race**			
Black or African American	94	97	1.02 (0.77, 1.36)
White	922	995	1.05 (0.96, 1.15)
Asian	57	57	1.19 (0.82, 1.72)

^*^Hazard ratio for outcomes among Psoriasis with type 2 diabetes group compared to Psoriasis without type 2 diabetes group subjects (after prosperity score matching).

### Psoriasis and smoking patients face a higher risk of psoriatic arthritis

**Figure 2:**
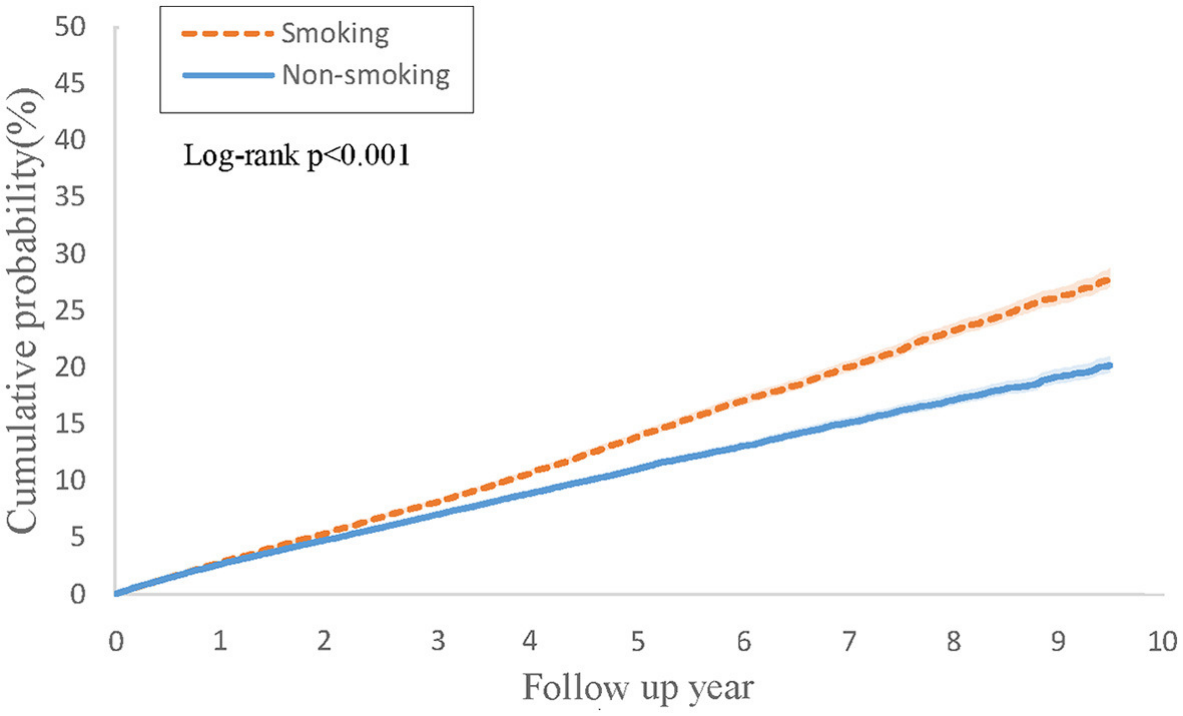
Kaplan-Meier curves of cumulative probability (%) of psoriatic arthritis comparing psoriasis with smoking history and without smoking history groups.

Table 3 illustrates the findings from Cox regression analysis for the initial group. In comparison to patients with PsO but without a smoking history, those with both PsO and smoking history exhibited an elevated risk of developing PsA (HR, 1.11; 95% CI 1.06–1.17), with the combined outcome demonstrating a heightened hazard ratio of 1.28 (95% CI 1.24–1.33). Figure 2 shows the Kaplan-Meier curves revealed a higher incidence of PsA in the group with both PsO and smoking history compared to the PsO group without smoking history (log-rank *P* < 0.001).

**Table 3:** Hazard ratio and 95% CIs for the risk of psoriatic arthritis.

	**Patients in cohort**	**Patients with outcome**	Hazard ratio^*^(95% CI)
**Composite End Points**			
**Risk of psoriatic arthritis**			
Psoriasis with smoking history	74,046	3,010	1.11 (1.06, 1.17)
Psoriasis without smoking history	74,046	2,568	Reference
**Risk of psoriatic arthritis or mortality**			
Psoriasis with smoking history	74,046	7,142	1.28 (1.24, 1.33)
Psoriasis without smoking history	74,046	5,344	Reference

^*^Hazard ratio for outcomes among Psoriasis with smoking history group compared to Psoriasis without smoking history group subjects (after prosperity score matching).

95% CI, 95% confidence interval.

### Psoriasis combined with type 2 diabetes and smoking face a slightly higher risk of psoriatic arthritis

**Figure 3:**
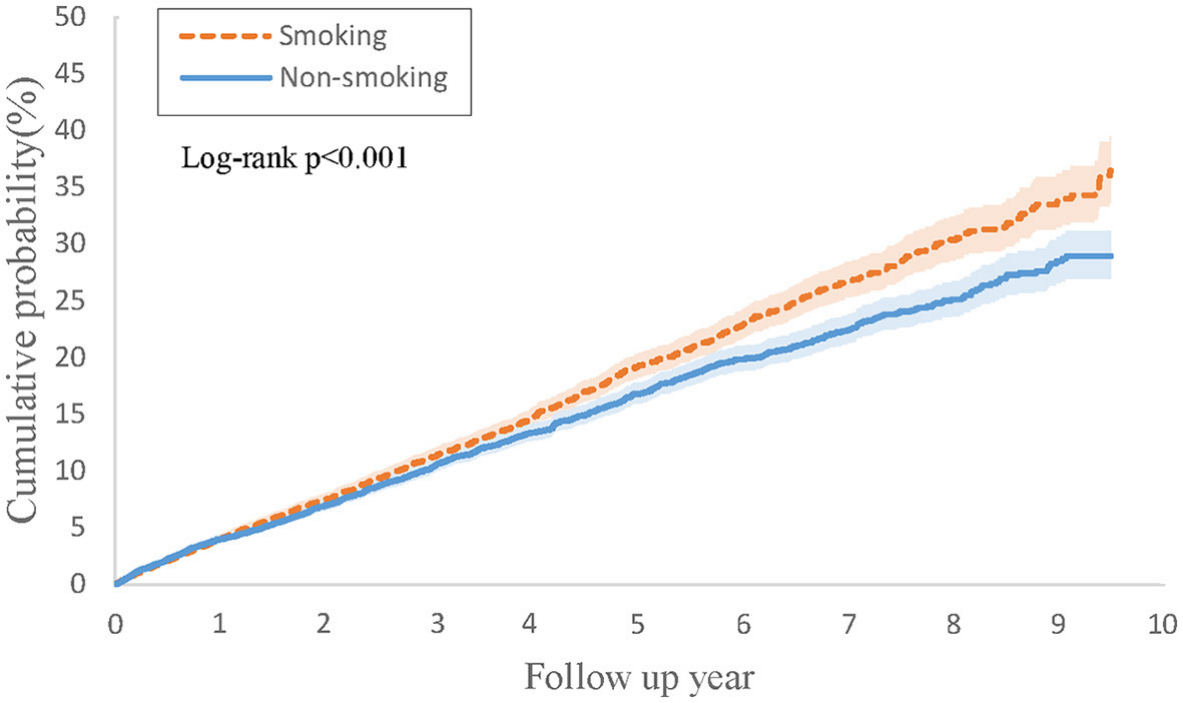
Kaplan-Meier curves of cumulative probability (%) of psoriatic arthritis comparing psoriasis and type 2 diabetes with smoking history and without smoking history groups.

Table 4 outlines the outcomes derived from the Cox regression analysis for this group. In contrast to individuals with PsO and type 2 DM but without a smoking history, those with both PsO and type 2 DM and a history of smoking were not found to be associated with an increased risk of developing psoriatic arthritis (HR, 1.05; 95% CI 0.92–1.20). However, the combined result revealed a higher risk of 1.15 (95% CI 1.06–1.23). Figure 3 depicts Kaplan-Meier curves, indicating that the group with a history of smoking, along with PsO and DM, experienced a higher incidence of psoriatic arthritis compared to the group without a smoking history (log-rank *P* < 0.001).

**Table 4:** Hazard ratio and 95% CIs for the risk of psoriatic arthritis.

	**Patients in cohort**	**Patients with outcome**	Hazard ratio^*^(95% CI)
**Composite End Points**			
**Risk of psoriatic arthritis**			
Psoriasis and type 2 diabetes with smoking	13,065	434	1.05 (0.92, 1.20)
Psoriasis and type 2 diabetes without smoking	13,065	425	Reference
**Risk of psoriatic arthritis or mortality**			
Psoriasis and type 2 diabetes with smoking	13,065	1,484	1.15 (1.06, 1.23)
Psoriasis and type 2 diabetes without smoking	13,065	1,355	Reference

^*^Hazard ratio for outcomes among Psoriasis with smoking history group compared to Psoriasis without smoking history group subjects (after prosperity score matching).

95% CI, 95% confidence interval.

## Discussion

With retrospective cohort data from the TriNetX database, our findings first revealed that PsO patients with type 2 DM exhibit a higher incidence rate of PsA than PsO patients without type 2 DM. Additionally, we showed that PsO patients who smoke also demonstrate an elevated incidence rate of PsA compared to non-smoking PsO patients. However, the impact of smoking seems less pronounced than that of type 2 DM in influencing the incidence of PsA. We further revealed that smoking does not considerably increase the incidence rate of PsA in PsO patients with type 2 DM. With these findings, we found that intrinsic factors outweigh external factors in PsA emergence in PsO patients and propose the hypothesis that genetic factors play a more substantial role in the occurrence of PsA in PsO patients compared to external factors such as smoking. In essence, the development of PsA in PsO patients appears predetermined rather than transitioning from PsO due to inadequate control of skin lesions.

Previous genome-wide association analyses of PsA and PsO uncovered distinct genetic architectures for these conditions (23). A recent genome-wide meta-analysis study found that the heritability of PsA is comparable to that of PsO; it identified a novel genome-wide significant susceptibility locus for PsA development on chromosome 22q11, and key pathways differentiating PsA from PsO, such as NF-κB and Wnt signaling, were identified (24). Caputo's review revealed that PsO and PsA exhibited distinct genes but shared some genetic factors (25). According to Mulder's systemic reviews, specific genetic markers demonstrated moderate evidence as potential risk factors for the onset of PsA in individuals with PsO. Notably, the presence of HLA-B^*^27 was higher in PsO patients who developed arthritis than those who did not, suggesting a possible differentiation capability for HLA-B^*^27 in identifying PsO patients with or without PsA. Additionally, the occurrence of a specific single nucleotide polymorphism (SNP) in IL-12 (rs2082412) was found to be lower in PsA patients compared to those with PsO (26). Zabotti's meta-analysis identified various risk factors for PsA in individuals with PsO, such as a family history of PsA, the severity of PsO, nail involvement, BMI, image-detected musculoskeletal (MSK) inflammation, and arthralgia (7). Image-detected MSK inflammation and arthralgia may not be considered risk factors for the development of PsA in PsO patients, as these symptoms could appear in PsA-confirmed patients. Exploring residual risk factors, it becomes evident that both genetic components and external triggers play roles in the development of PsA in PsO. Drawing on these genetic studies, risk factors, and our research findings, we propose that these two diseases possess distinct genetic backgrounds despite sharing a connection. Their courses may manifest differently, primarily due to internal rather than external factors, and this perspective underscores the notion that PsO and PsA are akin to brothers rather than twins.

The latest meta-analysis examining whether biological treatments for PsO reduce the risk of PsA reported positive results (27), contrary to Meer's opposing findings (28). In an animal study by Chen et al., PsA developed after the onset of PsO due to sustained IL-23 production (6). Consequently, it is suggested that PsA progressed from PsO, and early intervention with csDMARDs or bDMARDs, especially bDMARDs on IL-12/23 axial, is a hint to prevent the onset of PsA (8). However, it's important to note that animal study results may not necessarily align with human outcomes. Furthermore, Singla's research shows that, compared to tumor necrosis factor (TNF) inhibitors, IL-23 and IL-12/23 “inhibitor” may lower the incidence of PsA in PsO. Nevertheless, a closer examination reveals that in the IL-12/23 subgroup, the prevalence of diabetes and smoking is lower than in other subgroups. As our findings demonstrate that diabetes and smoking can increase the incidence of PsA in PsO patients, it's crucial to acknowledge that these variables were not adjusted, potentially influencing the study results. Additionally, csDMARDs and bDMARDs used in PsO patients have already demonstrated the effects on PsA (29). While these medications can temporarily alleviate symptoms and signs of arthritis, enthesitis, and dactylitis, their impact diminishes over time. Consequently, csDMARDs and bDMARDs can be observed to reduce the incidence of PsA in PsO patients, but they do not offer a permanent solution to prevent the occurrence of PsA.

The shift from PsO to PsA has long been assumed, promoting numerous attempts to identify the risk factors in PsO patients that would lead to the development of PsA. However, these efforts have yet to yield conclusive results (15). Suppose all PsA cases originated from PsO. In that case, it remains puzzling why only 30% of PsO patients eventually experience PsA, why ~10% of PsA cases emerge before the onset of PsO, and why PsA can still occur even after PsO lesions have been cleared through robust treatment with csDMARDs and bDMARDs. Our study results and proposed hypothesis offer a plausible explanation for the questions above.

The strengths of this study include its extensive inclusion of individuals from real-world settings, a 10-year follow-up period, comprehensive coverage of various csDMARDs and bDMARDs treatments for PsO and PsA, and the use of PSM to minimize bias from confounding factors. However, several limitations must be acknowledged.

Firstly, type 2 DM is categorized as an intrinsic risk factor and smoking as an extrinsic risk factor for PsA incidence in PsO patients. This approach is assertive but reasonably grounded in some evidence. Intrinsic factors are mainly related to genetics, yet genetic testing is not performed on every PsO patient, and no specific genetic marker is currently confirmed as a risk indicator for transition from PsO to PsA. We also considered including a family history of PsO or PsA in our analysis, but this information is only partially available in TriNetX. Apart from smoking, extrinsic factors might include dietary habits, alcohol consumption, and occupational exposure to chemicals. These data are also only partially available or analyzable in TriNetX. Therefore, we selected diabetes and smoking as the two variables we could reliably assess on TriNetX, representing extrinsic and intrinsic factors to ensure our results are relatively objective.

Secondly, BMI, PASI, and nail involvement in PsO are considered risk factors for transitioning from PsO to PsA. We did not include these variants for PSM, which may impact the results. Acosta's and Gisondi's reports showed different results regarding BMI as a risk factor for PsO transition to PsA (4, 5). Additionally, BMI information from TriNetX may need to be completed and fluctuate over time. PASI is not well-recorded in TriNetX, so it could not be considered a reliable variant for further analysis. Regarding nail involvement in PsO patients, ICD-10 coding for nail disorder is not specific to psoriasis and may not be coded in PsO patients, leading to underestimation and unreliability for analysis.

Thirdly, electronic medical record data may be susceptible to entry errors and data gaps, potentially leading to underreporting minor musculoskeletal symptoms and missing data, which could impact the results. This effect may diminish with a large number of patients and data. Additionally, outcomes outside the TriNetX network might need to be adequately captured during long-term follow-up. Furthermore, the analysis did not account for residual confounding factors such as csDMARDs and bDMARDs, which could alleviate or increase arthritis symptoms in PsA and were not considered in PSM. Consequently, these findings warrant further exploration and confirmation through robust methodologies and additional evidence.

In summary, our retrospective cohort study utilizing the TriNetX database revealed intrinsic factors outweigh externals in PsA emergence in PsO patients, and a review of genetic investigations on PsO and PsA revealed a genetic similarity but not identical between PsO and PsA. This led us to propose that PsA is not a mandatory evolutionary progression from PsO but is predominantly predetermined genetically. While csDMARDs and bDMARDs may postpone the onset of PsA in PsO patients, they do not entirely prevent it. Considering the inconclusive findings on risk factors for PsO patients to develop PsA, except for the musculoskeletal-related symptoms, signs, and various laboratory and imaging results, we recommend that further studies may focus on genetic disparities between PsO and PsA as potential risk indicators rather than relying solely on phenotypic distinctions.

## Data availability statement

The original contributions presented in the study are included in the article/Supplementary material, further inquiries can be directed to the corresponding author.

## Ethics statement

Ethical approval was not required for the study involving humans in accordance with the local legislation and institutional requirements. Written informed consent to participate in this study was not required from the participants or the participants' legal guardians/next of kin in accordance with the national legislation and the institutional requirements.

## Author contributions

A-PH: Conceptualization, Investigation, Writing – original draft. P-LL: Data curation, Formal analysis, Methodology, Software, Writing – original draft. P-YL: Resources, Writing – review & editing. JW: Supervision, Validation, Writing – review & editing.
