# Supplementary material for: From psoriasis to psoriatic arthritis: epidemiological insights from a retrospective cohort study of 74,046 patients
Source: Front Med (Lausanne). 2024 Jun 27;11:1419722. doi: 10.3389/fmed.2024.1419722 (PMC11236760; doi:10.3389/fmed.2024.1419722)
Supplement: Supplementary file 1 [file Table_1.DOCX]

**Supplementary tables**

Supplementary table S1. First cohort baseline characteristics

|  | Before PSM | | | After PSM | | |
| --- | --- | --- | --- | --- | --- | --- |
|  | psoriasis with  type 2 diabetes | psoriasis without  type 2 diabetes | SMD | psoriasis with  type 2 diabetes | psoriasis without  type 2 diabetes | SMD |
| **N** | 45,135 | 284,404 |  | 42,315 | 42,315 |  |
| Age at index (Mean±SD) | 55.0 +/- 17.6 | 43.3 +/- 20.2 | 0.618 | 54.5 +/- 17.7 | 56.1 +/- 17.6 | 0.09 |
| **Sex** |  |  |  |  |  |  |
| Female | 22484(49.8%) | 157286(55.3%) | 0.11 | 21225(50.2%) | 20765(49.1%) | 0.022 |
| Male | 22636(50.2%) | 127033(44.7%) | 0.11 | 21075(49.8%) | 21542(50.9%) | 0.022 |
| **Ethnicity** |  |  |  |  |  |  |
| Hispanic or Latino | 5004(11.1%) | 25809(9.1%) | 0.067 | 4534(10.7%) | 4290(10.1%) | 0.019 |
| Not Hispanic or Latino | 31310(69.4%) | 190904(67.1%) | 0.048 | 29076(68.7%) | 29404(69.5%) | 0.017 |
| Unknown Ethnicity | 8821(19.5%) | 67691(23.8%) | 0.103 | 8705(20.6%) | 8621(20.4%) | 0.005 |
| **Race** |  |  |  |  |  |  |
| American Indian or Alaska Native | 232(0.5%) | 1039(0.4%) | 0.022 | 211(0.5%) | 201(0.5%) | 0.003 |
| Asian | 1714(3.8%) | 10396(3.7%) | 0.008 | 1631(3.9%) | 1683(4%) | 0.006 |
| Black or African American | 4006(8.9%) | 17879(6.3%) | 0.098 | 3614(8.5%) | 3529(8.3%) | 0.007 |
| Native Hawaiian or Other Pacific Islander | 237(0.5%) | 838(0.3%) | 0.036 | 216(0.5%) | 246(0.6%) | 0.01 |
| White | 34444(76.3%) | 217651(76.5%) | 0.005 | 32223(76.2%) | 32349(76.4%) | 0.007 |
| Unknown Race | 4502(10%) | 36601(12.9%) | 0.091 | 4420(10.4%) | 4307(10.2%) | 0.009 |
| **Medical utilization** |  |  |  |  |  |  |
| Office or Other Outpatient Services | 23967(53.1%) | 102740(36.1%) | 0.347 | 21483(50.8%) | 21357(50.5%) | 0.006 |
| Hospital Inpatient Services | 2945(6.5%) | 5080(1.8%) | 0.239 | 2269(5.4%) | 1978(4.7%) | 0.032 |
| **Socioeconomic and psychosocial circumstances** | 6227(13.8%) | 7706(2.7%) | 0.411 | 4259(10.1%) | 3919(9.3%) | 0.027 |
| **Lifestyle** |  |  |  |  |  |  |
| Tobacco use | 7436(16.5%) | 8473(3%) | 0.468 | 5202(12.3%) | 4811(11.4%) | 0.029 |
| Nicotine dependence | 6839(15.2%) | 13100(4.6%) | 0.359 | 5174(12.2%) | 4712(11.1%) | 0.034 |
| Alcohol related disorders | 1818(4%) | 3739(1.3%) | 0.169 | 1388(3.3%) | 1227(2.9%) | 0.022 |
| **Comorbidities** |  |  |  |  |  |  |
| Hypertensive diseases | 18280(40.5%) | 30206(10.6%) | 0.729 | 15637(37%) | 15448(36.5%) | 0.009 |
| Hyperlipidemia | 14431(32%) | 24717(8.7%) | 0.604 | 12341(29.2%) | 12256(29%) | 0.004 |
| Asthma | 4374(9.7%) | 10398(3.7%) | 0.244 | 3254(7.7%) | 2980(7%) | 0.025 |
| Chronic obstructive pulmonary disease | 3087(6.8%) | 3641(1.3%) | 0.285 | 2120(5%) | 1975(4.7%) | 0.016 |
| Atrial fibrillation and flutter | 2131(4.7%) | 3265(1.1%) | 0.213 | 1735(4.1%) | 1588(3.8%) | 0.018 |
| Heart failure | 2893(6.4%) | 2230(0.8%) | 0.306 | 1993(4.7%) | 1235(2.9%) | 0.094 |
| Mental / dementia | 692(1.5%) | 1285(0.5%) | 0.109 | 513(1.2%) | 533(1.3%) | 0.004 |
| Chronic kidney disease | 3552(7.9%) | 3140(1.1%) | 0.331 | 2633(6.2%) | 2304(5.4%) | 0.033 |
| Anxiety disorders | 7835(17.4%) | 19337(6.8%) | 0.328 | 6305(14.9%) | 5125(12.1%) | 0.082 |
| Diseases of liver | 4646(10.3%) | 5661(2%) | 0.351 | 3188(7.5%) | 2777(6.6%) | 0.038 |

Note:

SMD: standardized mean difference.

*PSM (matching includes Age at index, Sex, Race, Ethnicity, Medical utilization, Socioeconomic, Lifestyle, and comorbidities.)

Supplementary table S2. Second cohort baseline characteristics

|  | Before PSM | | | After PSM | | |
| --- | --- | --- | --- | --- | --- | --- |
|  | psoriasis with  smoking history, | psoriasis without  smoking history | SMD | psoriasis with  smoking history, | psoriasis without  smoking history | SMD |
| **N** | 86,320 | 252,027 |  | 74,046 | 74,046 |  |
| Age at index (Mean±SD) | 45.2 +/- 19.3 | 45.1 +/- 20.6 | 0.001 | 45.4 +/- 18.8 | 46.2 +/- 19.3 | 0.041 |
| **Sex** |  |  |  |  |  |  |
| Female | 44814(51.9%) | 137784(54.7%) | 0.055 | 37891(51.2%) | 38002(51.3%) | 0.003 |
| Male | 40938(47.4%) | 111858(44.4%) | 0.061 | 35589(48.1%) | 35685(48.2%) | 0.003 |
| **Ethnicity** |  |  |  |  |  |  |
| Hispanic or Latino | 10259(11.9%) | 21934(8.7%) | 0.105 | 7786(10.5%) | 7653(10.3%) | 0.006 |
| Not Hispanic or Latino | 64795(75.1%) | 161773(64.2%) | 0.238 | 55244(74.6%) | 55364(74.8%) | 0.004 |
| Unknown Ethnicity | 11266(13.1%) | 68320(27.1%) | 0.356 | 11016(14.9%) | 11029(14.9%) | <0.001 |
| **Race** |  |  |  |  |  |  |
| American Indian or Alaska Native | 403(0.5%) | 876(0.3%) | 0.019 | 313(0.4%) | 308(0.4%) | 0.001 |
| Asian | 2185(2.5%) | 10453(4.1%) | 0.09 | 1921(2.6%) | 1857(2.5%) | 0.005 |
| Black or African American | 7359(8.5%) | 15209(6%) | 0.096 | 5938(8%) | 6186(8.4%) | 0.012 |
| Native Hawaiian or Other Pacific Islander | 321(0.4%) | 804(0.3%) | 0.009 | 287(0.4%) | 297(0.4%) | 0.002 |
| White | 70143(81.3%) | 186182(73.9%) | 0.178 | 59771(80.7%) | 59546(80.4%) | 0.008 |
| Unknown Race | 5909(6.8%) | 38503(15.3%) | 0.271 | 5816(7.9%) | 5852(7.9%) | 0.002 |
| **Medical utilization** |  |  |  |  |  |  |
| Office or Other Outpatient Services | 46005(53.3%) | 84733(33.6%) | 0.405 | 34927(47.2%) | 35057(47.3%) | 0.004 |
| Hospital Inpatient Services | 4198(4.9%) | 4347(1.7%) | 0.177 | 2771(3.7%) | 2746(3.7%) | 0.002 |
| **Socioeconomic and psychosocial circumstances** | 12494(14.5%) | 1693(0.7%) | 0.54 | 1636(2.2%) | 1693(2.3%) | 0.005 |
| **Comorbidities** |  |  |  |  |  |  |
| Hypertensive diseases | 20624(23.9%) | 31358(12.4%) | 0.3 | 14788(20%) | 15241(20.6%) | 0.015 |
| Hyperlipidemia | 15207(17.6%) | 26990(10.7%) | 0.199 | 11417(15.4%) | 11758(15.9%) | 0.013 |
| Diabetes mellitus | 12635(14.6%) | 14711(5.8%) | 0.293 | 7809(10.5%) | 6747(9.1%) | 0.048 |
| Asthma | 7250(8.4%) | 7959(3.2%) | 0.226 | 4169(5.6%) | 4258(5.8%) | 0.005 |
| Chronic obstructive pulmonary disease | 4805(5.6%) | 2337(0.9%) | 0.264 | 2381(3.2%) | 2112(2.9%) | 0.021 |
| Atrial fibrillation and flutter | 2090(2.4%) | 3653(1.4%) | 0.071 | 1525(2.1%) | 1402(1.9%) | 0.012 |
| Heart failure | 2964(3.4%) | 2646(1%) | 0.162 | 1557(2.1%) | 1467(2%) | 0.009 |
| Mental / dementia | 847(1%) | 1217(0.5%) | 0.058 | 481(0.6%) | 555(0.7%) | 0.012 |
| Chronic kidney disease | 3177(3.7%) | 4386(1.7%) | 0.12 | 2208(3%) | 2122(2.9%) | 0.007 |
| Sleep disorders | 10983(12.7%) | 10499(4.2%) | 0.311 | 6293(8.5%) | 5041(6.8%) | 0.064 |
| Diseases of liver | 6046(7%) | 4801(1.9%) | 0.249 | 3153(4.3%) | 3147(4.3%) | <0.001 |

Note:

SMD: standardized mean difference.

*PSM (matching includes Age at index, Sex, Race, Ethnicity, Medical utilization, Socioeconomic, Lifestyle, and comorbidities.)

Supplementary table S3. Hazard ratio and 95% CIs for the risk of psoriatic arthritis by Global Collaborative Network

| Outcome | Patients  in cohort | Patients with  outcome | Hazard ratio*  (95% CI) |
| --- | --- | --- | --- |
| Composite end points |  |  |  |
| Risk of psoriatic arthritis |  |  |  |
| Psoriasis with type 2 diabetes | 45,659 | 1,336 | 1.09(1.01, 1.18) |
| Psoriasis without type 2 diabetes | 45,659 | 1,357 | Reference |
| Risk of psoriatic arthritis or mortality |  |  |  |
| Psoriasis with type 2 diabetes | 45,659 | 4,275 | 1.32(1.26, 1.38) |
| Psoriasis without type 2 diabetes | 45,659 | 3,648 | reference |

*Hazard ratio for outcomes among Psoriasis with type 2 diabetes group compared to Psoriasis without type 2 diabetes group subjects (after prosperity score matching).

95% CI, 95% confidence interval
